# A Framework for Learning Event Sequences and Explaining Detected Anomalies in a Smart Home Environment

**DOI:** 10.1007/s13218-022-00775-5

**Published:** 2022-10-31

**Authors:** Justin Baudisch, Birte Richter, Thorsten Jungeblut

**Affiliations:** 1 Faculty of Engineering and Mathematics, Bielefeld University of Applied Sciences, Bielefeld, Germany; 2 Medical Assistance Systems, Medical School OWL, Center for Cognitive Interaction Technology (CITEC), Bielefeld University, Bielefeld, Germany; 3 Faculty of Engineering and Mathematics, Bielefeld University of Applied Sciences, Bielefeld, Germany

**Keywords:** Ambient assisted living, Anomaly detection, Internet of things, Explainable AI, 68T01, 68T05

## Abstract

This paper presents a framework for learning event sequences for anomaly detection in a smart home environment. It addresses environment conditions, device grouping, system performance and explainability of anomalies. Our method models user behavior as sequences of events, triggered by interaction of the home residents with the Internet of Things (IoT) devices. Based on a given set of recorded event sequences, the system can learn the habitual behavior of the residents. An anomaly is described as deviation from that normal behavior, previously learned by the system. One key feature of our framework is the explainability of detected anomalies, which is implemented through a simple rule analysis.

## Introduction

The demographic change is leading to an increase in the number of elderly people. This results in an increased number of chronic illnesses and multimorbidity leading to a need for medical care services [1]. In 2019, 4.13 million people needed care services in Germany. Around four out of five people are cared for at home. Most of the care is done by relatives, often supported by a nursing service. The workload of nurses recently steadily increased in all areas – not only due to the COVID-19 pandemic [2]. Cognitive systems can simplify and secure the life of the persons in need of care, and can also decrease the workload of relatives and/or the nursing staff (caregiver), e.g. by using artificial intelligence for tour planning, anomaly detection or intelligent emergency call systems. Smart home technologies in particular offer a wide range of options here to support the persons in need of care, and their caregivers. Anomaly detection could help here in two ways. Firstly, acute anomalies in daily routines can be detected (e.g. person goes to the bathroom but cannot get out) and could make an emergency call. In addition, they can provide helpful insights into the behavior of the person to be cared for that may have changed due to illness (e.g. search behavior due to the onset of dementia) and so caregivers can intervene at an early stage. Explainable AI methods can thereby provide meaningful explanations to these end users about smart home operations [3].

This paper is structured as follows: In the next section, we’ll cover different anomaly detection techniques based on user behavior, as well as some approaches to explain those anomalies. Then we’ll explain the platforms used to explore our own approach. After that, we’ll dive deeper into the system implementation, where we cover the learning technique, anomaly detection and how to explain those anomalies. In the end, we’ll give a short summary of what has been done and what is to be done in the future.

## Related Work

Anomaly detection is used in various contexts like image detection, data cleaning or identifying flaws in manufactured materials [4]. Common approaches are based on multidimensional data and, referred to as *point-based anomaly detection* [5], do not consider the sequence of discrete events.

In this work, we’ll focus on techniques which handle discrete event sequences. A common approach for this, called Distance Based Anomaly Detection (DBAD), is a clustering-technique where a distance-based criterion is used to detect outliers (anomalies) [4]. A first method was introduced in [6], where a One Class Support Vector Machine (OCSVM) was used to transform data points into a feature space, where points close to the origin were identified as anomalies. Another approach is shown by [7], where a Self-organizing Map (SoM) was used to classify user activities by their venue and duration. An anomaly was then detected, if a new data point wasn’t bounded to any cluster or excessively deviates from it.

In contrast to DBAD, there are probabilistic approaches. [8] introduced an algorithm to draw upon the temporal nature of sensor data collected in a smart home environment, where events were connected through temporal relationships like *A before B*, *C after D* and *X during Y*. With that, the algorithm learned the probability for each relation of event *A* and *B* for all existing events in the training data (*P*(*A*)). An event was then detected as an anomaly, when the counter-probability ($1 - P(A)$) exceeded a predetermined threshold.

Different from that, Yamauchi et al. presented a method which learned event sequences for different conditions [9]. For that, they defined an event sequence as events occurring within a time frame of *T* seconds from a previous event. A condition is described as a combination of time of day and sensor measurements like temperature or humidity. Each sequence was modeled in a tree data structure, where each node corresponds to one event. To erase potential noise and extract the essential event sequence, they generated event sequences from the monitored ones by building all possible permutations of them. To check whether an event belongs to a sequence or not, they used the timing information *T* between the events. In general, they presented an algorithm which tries to maximize the number of events in a sequence by continuously increasing *T*, without overfitting it. For anomaly detection, their algorithm tries to include a new event into a sequence and matches it against all previously learned sequences. If no match was found, the event was treated as an anomaly. In a newer version [10], Yamauchi et al. improved their implementation of the noise elimination algorithm. Therefore, they introduced a binary variable *v* “which indicates the events included in the generated sequence” [10] by the *i*th bit ($1 = included$, $0 = not\ included$).

A lack of research exists, considering the explainability of smart home operations to end-users [3]. In [4], Davidson described an explanation approach for minimum likelihood threshold anomaly detection, where an observation was anomalous, when it doesn’t belong to a cluster with a likelihood greater than some threshold. He did so, “by considering the most probable class and determining what changes to the observation could have increased the class likelihood above this threshold” [4]. In a different context, Lim et al. found out that, especially *why*, explanations can improve user’s understanding and trust in intelligent systems [11].

Although there is a lot of research on anomaly detection in smart homes, a research gap exists in the field of the explanation of such anomalies. Therefore, we propose a new approach for anomaly detection, which (i) uses environment conditions and device grouping (ii), is deployed in three real-world smart homes and (iii) provided a visual and textual explanation.

## Research Platform

Our work was carried out within the *KogniHome e.V.* [12] in Bielefeld, Germany, and a private household (*H1*) with more than 200 IoT devices. The *KogniHome e.V.* is a non-profit association based on an interdisciplinary network of partners from research, industry and care, with the main goal to develop user-oriented Ambient Assisted Living (AAL) solutions for the demographic change. As it is shown in the next section, these two scenarios can be divided into different levels of complexity. The *KogniHome e.V.* research apartment has a low and *H1* a high complexity.

### Setup

The research apartment *KogniHome* based on  [13] acts as a reference system with six rooms: Corridor, Bathroom, Living Room, Kitchen, Bedroom, and a Balcony. Each room, except the Balcony, is equipped with a motion sensor. The windows in bed- and bathroom, as well as the door to the balcony, are equipped with magnetic contact switches for state detection (*open* or *closed*). Three smart power plugs with integrated power sensors in the kitchen are connected to a coffee machine, toaster and water heater. Obviously, those gadgets could be smart by themselves, but the current goal of the *KogniHome e.V.* is to convert a non-smart home into a smart home by cheap and easy-integrable IoT devices to be affordable by everybody. Furthermore, several sophisticated research prototypes have already been implemented in the *KogniHome*: an intelligent cooking assistant [14], a connected chair [15], an intelligent door/wardrobe [16] and a smart mirror [16].

*H1* is a two-story house with 14 rooms plus the hallway. It embeds more than 200 IoT nodes with more than 500 sensor and actor endpoints. Devices range from unobtrusive sensors up to advanced systems like cleaning robots. For anomaly detection we use simple binary sensors (on/off, open/closed, etc.) like PIR motion sensors, smart switches and door and window contact sensors as well as more complex sensors like smart thermometers or luminance sensors to track, i.e. the outside temperature and brightness. The communication is based on a heterogeneous set of wireless standards including Z-Wave, ZigBee and Wi-Fi.

## Definitions

### Definition 1

An **event** is described as the state-change of a IoT device (i.e. off to on) on a specific timestamp under consideration of its environmental conditions.

### Definition 2

An **environmental condition** is anything that influences an event. A simple example is the temperature. Turning on the heater in the winter (cold) is completely normal. Instead, turning on the heater in the summer (hot) is not. So, the temperature is affecting the frame of an event and therefore must also affect the output of the anomaly detection.

### Definition 3

An **event sequence** is a series of events connected to each other. We differentiate between two types of sequences, the raw sequence and the merged sequence. The raw sequence is directly derived from the sensor events. Multiple raw sequences can then be merged into each other, as described later.

### Definition 4

An **anomaly** is defined as a raw sequence, which is a deviation from the learned normal behavior of the user.

## System Implementation

In this section, we describe the method used to detect anomalies, what type of anomalies we can detect with this approach and how to explain these to the smart home user.^1^

### Learning Model

Based on [9], our system model consists of a set of events (monitored behavior of the user(s)), annotated with one or more environmental conditions, like time of day or temperature. In addition, our model allows for the pre-grouping of IoT devices, i.e. by their location. This is especially useful in larger sensor environments like *H1*, to cope with multiple users simultaneously interacting in one household, and to prevent interference through known unrelated devices like environmental sensors (humidity, luminance etc.). The grouping is handled by the user and therefore is not limited to any requirements. Each device can have multiple groups. To store the event sequences, we use a Directed Acyclic Graph (DAG) instead of a tree structure as used in [9]. This increases the performance of the system, which will be explained in detail in the next section. The edge-weights of the sequence graph represent the number of occurrences of two subsequent events. These edge weights are initialized with ’1’ and increased during each merging step of the sequence generation.

### Sequence Generation

**Fig. 1:**
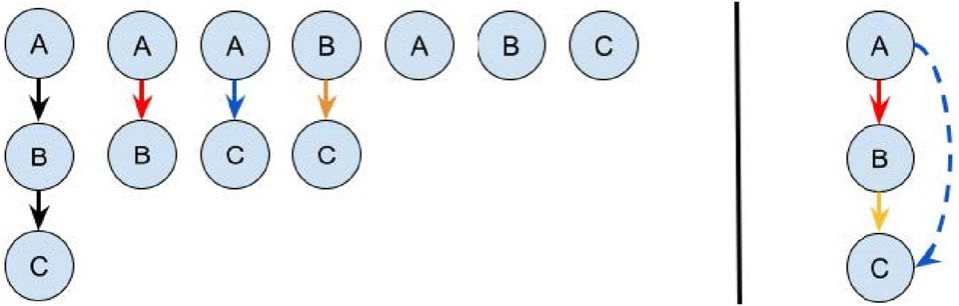
Explicit sequence generation after [9] (left) vs. our implicit sequence generation (right)

In [9] they used an explicit sequence generation algorithm, which means, in this context, that the sequences were generated subsequently from a previously recorded sequence by building up all possible combinations. We modified this approach by generating all possible sequences during recording. Therefore, each added event was connected to all existing predecessor events in the currently created sequence. This implicitly generates all possible combinations of the recorded event sequence. With this, we save a lot of memory used by the system. Figure 1 shows the sequence generation of [9] compared to our approach.

Additionally, in comparison to [9], we do not allow duplicate events in one sequence. Instead, in case of the occurrence of duplicate event, we terminate the current sequence and start a new one.

**Fig. 2:**
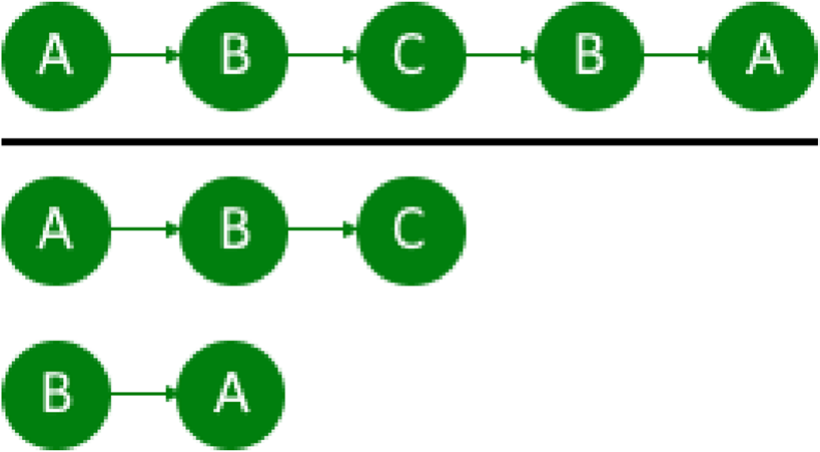
Sequence splitting on a duplicate event (here B) to get more generalized sequences

We made this restriction because, by this, we can generalize the output sequences and divide very large sequences into smaller ones. For example, if the user goes back and forth from the living room to the bathroom, this activity is split up into single forth- and back-activity sequences (Fig. 2). In [9], this would be one large sequence, preventing the system to merge following subsequences to this activity.

This step did not lead to an underestimation of the number of sequences. In early development stage, using the approach of [9], we discovered memory exhaustion with high numbers of sequences. To address this, we included a merging step into the algorithm, which merged equal or subsequences into one, by increasing the respective edge-weight of the DAG instead of storing all sequences separately.

Before explaining our learning approach in detail, we shortly dive into the mechanism of sensor grouping. As we implemented the approach of [9], it turned out that event sequences were often mixed up with unrelated events, especially due to IoT devices in the household update their status regularly. Another cause of the mixing of events are multiple-user environments, where two residents simultaneously act with the system in different rooms. To avoid these flaws, we not only added a whitelist-mechanism but also the possibility to group devices. Each individual sensor of an IoT can be assigned to one or more group. For example, this can be used to create activity sectors.

### Sequence Learning

With some algorithmic adjustments, our approach is based on the learning method of [9]. The system learns a timing parameter *T* which defines the maximum time allowed to elapse between the events within an event sequence. In [9], this *T* was globally used for every sequence. Unfortunately, this approach did not lead to satisfying results. In our real-world scenario, we contend fast and slow activities like walking from A to B or cooking. So, a globally defined *T* for all event sequences is not useful. To improve this, *T* is individually learned for every group. This does not conquer the problem of mixed (slow and fast) sequences within a group.

### Anomaly Detection

Our anomaly detection is shown in Algorithm 1–3. The basic concept of it is as follows. First, we create empty sequences for each group. After that, every monitored event is assigned to the sequences corresponding to its group. If the assignment fails due to a timeout, the sequence is considered as complete and needs to be analyzed for an anomaly. This is done via comparison against all learned sequences. To do so, Algorithm 2 tries to find one learned event sequence, which matches the monitored one. If it finds a match, the monitored sequence is normal behavior and vice versa an anomaly. To not be an anomaly against a learned sequence (*L*), the monitored sequence (*M*) needs to be a sub-graph of the learned one (all edges of *M* must exist in *L*). Also, every edge of *L* must-have a weight greater than a minimum-weight-threshold *W*, to consider rare-occurring edges as anomalies, too.
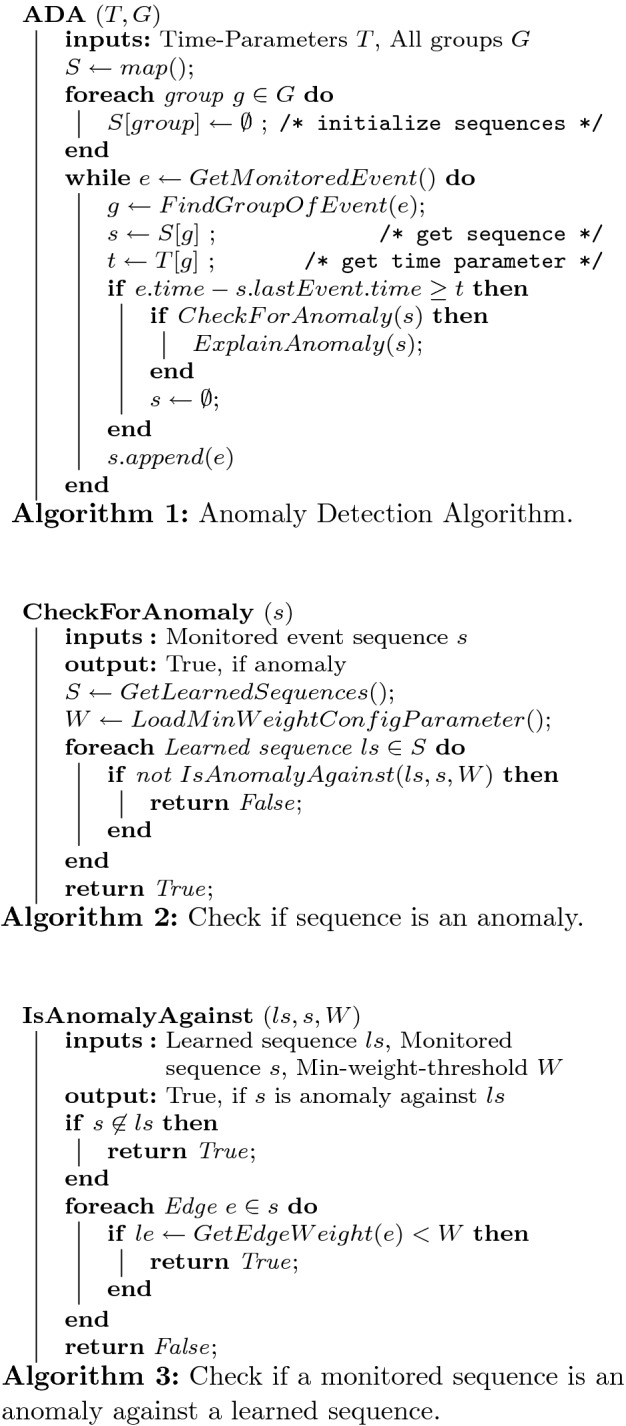


### Explanation

As the learning algorithm and the anomaly detection are unsupervised, it is important to give the user (e.g. a caregiver) an explanation of detected anomalies to fully understand decisions of the system and to evaluate the current situation of the home and the resident. With a detailed explanation, the user can better understand why the system suspects that it has detected an anomaly. Even if the system cannot describe the exact reason for an anomaly, it can nevertheless give the user important information for further tuning the performance of the system.

In the following section, we will describe what types of anomalies we can detect and explain with the described approach. For this, we will look on a simple example, where the algorithm learned the sequence given in Fig. 4. It’s a simple morning routine consisting of two motion events (living- and bedroom) and two power events (coffee machine and toaster). With the model described in 5.1, we can detect three types of anomalies:

1. Unknown event sequence
2. Insufficient weight
3. Wrong condition

**Fig. 3:**
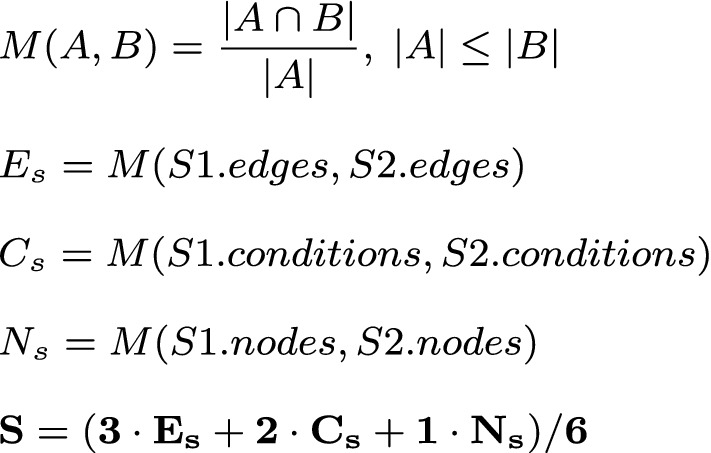
Similarity (*S*) of sequences *S*1 and *S*2

**Fig. 4:**
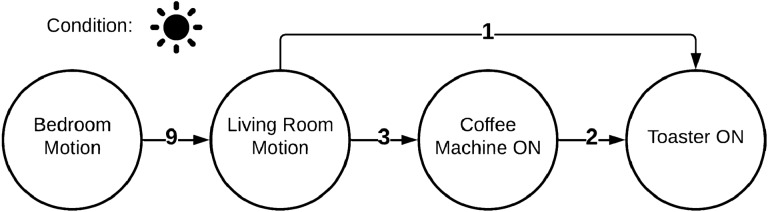
Event sequence: morning routine

If an event sequence is completely **unknown** to the system (R1), i.e. when the event-order is mixed (Fig. 4), the explanation component cannot find a reasonable explanation for the anomaly. Instead, the system tries to find the most equivalent event sequence for it. For this, a *similarity value* is defined (cf. Fig. 3). This similarity is a weighted value that indicates, how equal an event sequence *A* is to *B* - considering three parts of those sequences: nodes, edges and conditions. The equality (*M*(*A*, *B*)) we used in this work is stands in contrast to the Jaccard-coefficient [17]. This is because we do not consider the similarity in both directions (*A* to *B* and *B* to *A*). More, we just consider one direction (*A* to *B*), which means, how much of *A* exists in *B*. Although no direct explanation (except that the event sequence is unknown) can be provided, the system can give the best matching sequence so that the user can evaluate the current situation. **Insufficient weighting** (R2) of the sequence can be easily recognized. Normally, the system looks for an event sequence (matching the possible anomaly) in the normal behavior which edges exceed the user-defined minimal-weight-threshold. If it doesn’t find one, this would normally lead to *Unknown behavior* classification. During the explanation part, the system instead overrides this minimal-weight-threshold with 0. Then it rechecks whether the recorded sequence is matching any of the (now zero-weighted) sequences of the normal behavior. If the system this time finds a matching sequence, the anomaly was caused by an insufficient weighting (Fig. 6). The same principle is applied when checking for a **wrong condition** (R3). The condition of the event sequence is temporarily ignored, and the system considers every possible combination of conditions and applies it to the recorded event sequence. Then it checks whether another condition dissolves the anomaly. If this is the case, the system can specify a wrong condition as the reason for the anomaly (Fig. 7). In addition, the system can also describe, which condition would dissolve the anomaly.

**Fig. 5:**
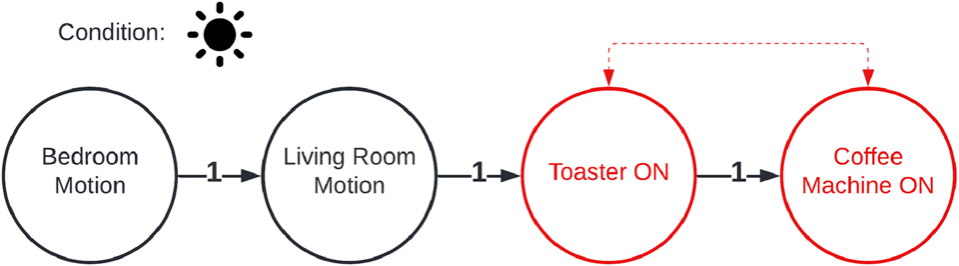
Anomaly: unknown event sequence

Textual explanation of anomaly in Fig. 5:

- The sequence is unknown by the system
- Reached similarity score 0.83 (max 1.0)
- 100% event similarity
- 66% event transition similarity
- 100% condition similarity

**Fig. 6:**
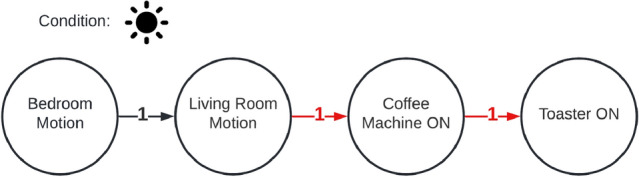
Anomaly: insufficient weight

Textual explanation of anomaly in Fig. 6.

- Found a matching event sequence, but the weights were to low

**Fig. 7:**
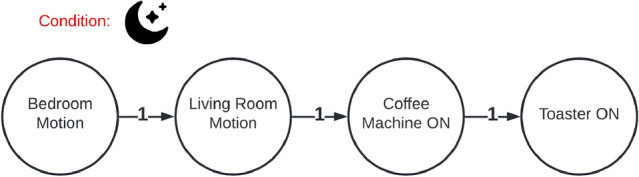
Anomaly: wrong condition

Textual explanation of anomaly in Fig. 7.

- The conditions of the event sequence are unknown by the system
- The event sequence is known by the system, but the conditions did not match any of the known
- Changing conditions NIGHT to MORNING, would make disappear the anomaly

## Real-World Deployment and Evaluation

As described in Sect. 3, we deployed the system in three different scenarios. In the *KogniHome* scenario, we grouped all sensors in one group. This is not recommended for higher scenarios like *H1*, because this can cause a mixture of unrelated interaction sequences. For simplicity, we decided to produce a one-way scenario in the *KogniHome*, shown in Fig. 4. The system extracted 9 event sequences and successfully merged these into 1 sequence. Then we triggered the anomalies shown in Figs. 5, 6 and 7 and the system could successfully identify and explain those anomalies. The textual explanations of the anomalies, given by the system, are shown below the affiliated figures. In the more complex *H1*-scenario 34,888 total events were recorded within a measurement period of 17 days. Events were separated into the following 6 groups: entrance, corridors, garden, basement, kitchen, bedroom. The learning algorithm produced the following outputs for a learning interval of 16 days (Table 1).

**Table 1:** Amount of generated event sequences in *H1*-scenario before and after merge, based on calculated T parameter

Group	T	#Sequences	#AfterMerge
Entrance	42 s	852	111
Corridors	72 s	1639	79
Garden	4 s	64	20
Basement	90 s	580	115
Kitchen	4 s	103	12
Bedroom	106 s	2403	209

The data of the 17th day was then used as a test set for the anomaly detection. It is important to mention, that day 17 did not include any real anomalies. It is a normal day like the 16 days before. Instead, the goal was to see, if the algorithm generates false-positive anomalies and how many of them. This was our evaluation criteria. Based on this setup, the algorithm produced the following anomalies (Table 2).

**Table 2:** Number of anomalies (U = unknown behavior, I = insufficient weighting, C = wrong condition) and None-Anomalies (#S) detected on the 17th day of the data set

Group	U	I	C	#A	#S
Entrance	0	0	0	0	14
Corridors	0	0	0	0	54
Garden	0	0	0	0	0
Basement	0	3	3	6	14
Kitchen	6	0	0	0	0
Bedroom	0	6	2	8	62

**Fig. 8:**
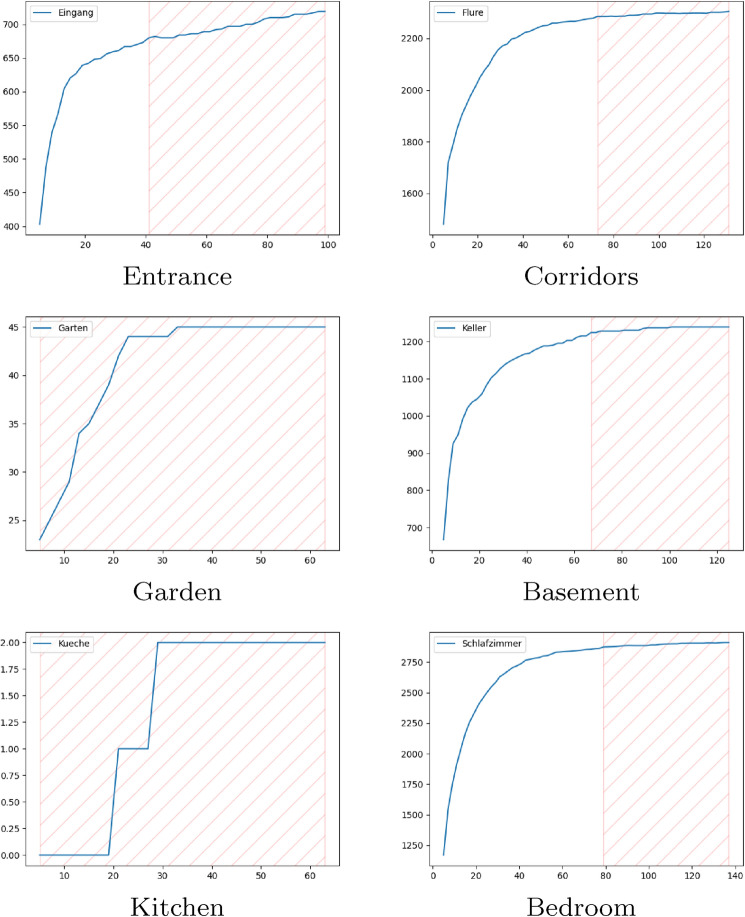
Number of pairs of event sequences of each group in *H1* plotted against T parameter, showing the termination criterion of T (see Table 1)

Table 2 shows that the algorithm does not find any false-positive anomaly in the *Unknown behavior* class, except for the *Kitchen*. This is because the learning algorithm could not extract enough event sequences from the *Kitchen* events (Fig. 8: *Kitchen*), for example, through an insufficient number of sensors. *Bedroom* and *Basement* both contain anomalies detected by *insufficient weighting* and *wrong condition*. This is most likely due to a short learning period.

To evaluate the provided explanations, we made a user study with 20 subjects in the age of 18–50. Therefore, we created a survey to analyze the usefulness of the textual explanations. To do so, the subjects first needed to classify given anomalies based only on their visual graph representation. For this, we provided four possible answers containing the three anomalies (R1–R3) and also the option that none of these match (multiple selection was possible). Then the subjects needed to classify given anomalies again, but regarding the textual explanation of the system. Table 3 shows the results of the study. In the survey, we handed out three different anomalies (A1–A3) to the user. A1, which was *unknown behavior*, A2 which was *insufficient weighting* and A3, which was *insufficient weighting* and *wrong condition* combined. As we can see, the combination of two anomalies classes lead to an incorrect assessment. Most of the subject selected only one of the two classes, but only one third selected both classes. The explanation made it even worse. This is, because the current version of the system could not explain multiple anomaly classes. Instead, the system only provided the *insufficient weighting* explanation. This obviously made the subjects uncertain, and four of them changed their opinion to only select the *insufficient weighting* classification. In contrast, the explanation of A1.1 increased the accuracy in consideration to A1. Even if the result of A3.1 increased the incorrectness of the answers, we can see how relevant the explanation can be to the user and what impact it has for the decision-making.

**Table 3:** Amount of correct and wrong classification of anomalies (A1, A2, A3 without, and A1.1, A2.1, A3.1 with explanation)

Anomaly	Correct	Wrong
A1	14	6
A1.1	18	2
A2	17	3
A2.1	17	3
A3	6	14
A3.1	2	18

## Conclusions and Future Work

Our evaluation shows, that the approach of [9] works for simple scenarios with only few sensors. The termination criterion for the learning of sequences is based on the number of pairs given in the sequences. We improved this approach by sensor grouping to avoid interferences of unrelated interactions. This is useful, as long as the sensors inside a group have similar timing conditions. If the group mixes slow- and fast-activity sensors, the algorithm either over- or underestimates the problem. For example, this is the case when we group motion sensors with kitchen sensors. The motion sensors are activated on a relatively fast time base. Operating appliances in the kitchen is comparatively slow. To conquer this problem, we started to explore new abort criteria for the sequence learning algorithm. Figure 8 visualized the current approach, by plotting the number of pairs (y-axis) against the time-frame (x-axis) for each group of *H1*. The hatched section of each plot shows the current termination criterion, where the number of pairs did not change significantly over a time span of 60 seconds. As we can see in the garden and kitchen plot, it isn’t a good choice to have one global criterion for all groups, especially when the number of sensors differs a lot. Also, we currently face the problem of many false positive anomalies due to lack of training data. In future, this will be handled through a feed mechanism, which allows the caregivers to validate found anomalies and back-propagate it to the system, improving it incrementally. Future work will therefore explore an algorithm, which not only learns the time-frames of each group, but also automatically learns a potential grouping and their abort criteria. Furthermore, we currently develop a visualization tool which allows us to track different stages of the algorithm.

## Footnotes

1 The source-code of the system can be found under https://github.com/jbaudisch/sharly_pub.
